# A fly model of SCA36 reveals combinatorial neurotoxicity of hexanucleotide and dipeptide repeats

**DOI:** 10.1371/journal.pgen.1011954

**Published:** 2025-12-03

**Authors:** Cheng-Tsung Hsiao, Ssu-Ju Fu, Ting-Ni Guo, Chia-Chi Lin, Yu-Jung Tsao, Wenying Chang, Yi-Chu Liao, Masayuki Hashimoto, Shu-Yi Huang, Yi-Chung Lee, Chien-Hung Yu, Chih-Chiang Chan

**Affiliations:** 1 Graduate Institute of Physiology, National Taiwan University, Taipei, Taiwan; 2 Department of Neurology, National Yang Ming Chiao Tung University School of Medicine, Taipei, Taiwan; 3 Department of Neurology, Neurological Institute, Taipei Veterans General Hospital, Taipei, Taiwan; 4 School of Medicine, Fu‑Jen Catholic University, New Taipei City, Taiwan; 5 Institute of Basic Medical Sciences, College of Medicine, National Cheng Kung University, Tainan, Taiwan; 6 Brain Research Center, National Yang Ming Chiao Tung University School of Medicine, Taipei, Taiwan; 7 Institute of Molecular Medicine, College of Medicine, National Cheng Kung University, Tainan, Taiwan; 8 Department of Medical Research, National Taiwan University Hospital, Taipei, Taiwan; 9 Department of Biochemistry and Molecular Biology, College of Medicine, National Cheng Kung University, Tainan, Taiwan; Duke-NUS: Duke-NUS Medical School, SINGAPORE

## Abstract

Spinocerebellar ataxia type 36 (SCA36) is a neurodegenerative disease caused by expanded (GGCCTG)n hexanucleotide repeat sequence in the *NOP56* gene. While the expanded repeats could transcribe and form toxic RNA foci within neurons, recent evidence indicates that translation of these repeats produces dipeptide repeats (DPR) that contribute to neurotoxicity. The relative impact of hexanucleotide RNA repeats (HRR) and DPR on the neurodegeneration of SCA36 remains unclear. Here, we established a *Drosophila* SCA36 model to dissect the neurotoxic effects of HRR and DPR. The fly model recapitulates the cellular defects observed in SCA36 patient fibroblasts, validating its relevance for mechanistic study of SCA36. Further engineering the transgenes to express individual DPRs reveal Proline-Glycine-DPR (PG-DPR) as the most potent neurotoxin causing progressive motor and sensory dysfunction. Expressing a series of the SCA36 transgenes with varying HRR lengths demonstrates an age- and length-dependent adult-onset neurodegeneration. Interestingly, sequence modification of the transgenes to exclusively express HRR or DPR alone causes a milder phenotype, indicating both HRR and DPR contribute partially to the pathogenicity of SCA36. Therefore, this model provides a valuable platform for screening drug targeting either HRR- or DPR-mediated toxicity of SCA36. Suppression of the RNA elongation factor *SUPT4H1* ortholog reduces RNA foci in cell culture. However, expression level of *SUPT4H1* was not changed in SCA36 patient cells. Interestingly, knockdown of the *Drosophila SUPT4H1* ortholog or 6-azauridine treatment to suppress RNA transcription aggravates the neurodegenerative phenotypes in both the fly models and patient-derived fibroblasts, highlighting the complex interplay of pathomechanisms in SCA36. These results underscore the need for carefully evaluating the potential side effects when designing therapeutic interventions for SCA36.

## Author summary

Tandem repeat expansions in the human genome are linked to several neurological diseases, yet the precise mechanisms through which these repeats lead to neural cell damage remain unclear. In our study, we explored this question by focusing on spinocerebellar ataxia type 36 (SCA36), a progressive disorder caused by an expanded repeat sequence in the *NOP56* gene. People with SCA36 experience imbalance, dyscoordination, and motor neuron degeneration. To investigate how the expanded repeats exert their neurotoxic effects, we generated a fruit fly model that recaptures key features seen in patients. Our findings show that both the repeated RNA and the dipeptide products derived from the repeat sequence contribute to neurotoxicity. Interestingly, when either component was expressed on its own, the deteriorated effects were milder, suggesting a combined toxic mechanism. Surprisingly, strategies aimed at reducing RNA production by manipulating the RNA elongation machinery worsened disease features, pointing to a complex disease process of SCA36. Our model not only helps clarify how repeat sequences influence the nervous system, but also serves as a platform for testing therapies. This work deepens our understanding of spinocerebellar degeneration and motor neuron disease and highlights the importance of cautious therapeutic development in repeat expansion disorders.

## Introduction

Spinocerebellar ataxia type 36 (SCA36, MIM:614153) is an autosomal dominant neurodegenerative disease caused by an expansion of a hexanucleotide repeat (GGCCTG)n (hereinafter referred to as (G3C2T)n) within the first intron of *NOP56* (NM_006392) gene. This expansion leads to various neurological symptoms, including progressive ataxia, dysarthria, sensorineural impairment, and muscle weakness, typically manifesting in adulthood of the patients [1–3]. The *NOP56* loci consists 3–14 repeats for unaffected individuals, while the patients affected by SCA36 carry repeats ranging from 30 to 2500 [1–5]. The association between the length of the repeat expansion and the clinical presentations of SCA36 patients remains to be elucidated. Although lack of comprehensive epidemiology investigation, SCA36 has been identified in multiple populations, particularly in Japan, Spain, and to a lesser extent in Taiwan, France, China, the United States, Australia, the United Kingdom, and Canada and other countries [1–10].

Recent studies have shed light on the potential mechanisms underlying SCA36 pathology. One major hypothesis suggests that the expanded hexanucleotide RNA repeats (HRR) form toxic foci within neurons and disrupt normal cellular functions [11,12]. Modulation of RNA elongation machinery may reduce HRR production and may serve as a potential therapeutic strategy for repeat expansion disorders such as SCA36 [12–14]. Supporting this hypothesis, studies in cell culture showed that inhibition of RNA elongation factor *Spt4* could reduce RNA foci and rescue the cellular derangement [12]. However, whether inhibiting RNA elongation could lead to neuroprotection against SCA36 toxicity in a living organism remains to be determined.

Alternatively, research suggests that the expanded repeats might be translated into dipeptide repeat proteins (DPR) through upstream open reading frame (uORF)-mediated translation or non-canonical translation mechanisms. These DPR have been observed in the cells and tissues obtained from affected patients and are thought to contribute to neurotoxicity as shown in other neurological disorders of similar repeat expansion, including the *C9ORF72* (GGGGCC)n related FTD/ALS (C9-ALS) [15–19]. However, a key question remains: what is the relative impact of HRR and DPR on SCA36 neurodegeneration?

To bridge the knowledge gap in SCA36 pathogenesis, we developed a model in the fruit flies *Drosophila melanogaster*. The model consists of a series of modified (G3C2T)n transgenes, allowing for utilizing the versatile and powerful fly genetics to characterize the relative contribution of HRR or DPR to disease severity. The phenotypes of the SCA36 flies recapitulate the cellular defects of the fibroblasts derived from SCA36 patients. Finally, as a proof-of-principle, we utilized the fly SCA36 model to explore potential therapeutic strategies for this disease.

## Materials & methods

### *Drosophila* strains and genetics

*Drosophila* stocks were maintained at 25°C on standard medium and genetic crosses were set up using standard fly husbandry practices. Details on fly strains and genotypes used in experiments are provided in Table 1 and on FlyBase (flybase.org), unless otherwise specified.

**Table 1:** Fly strains used in this study.

Strains	Source	Identifier	Figure
*D. melanogaster: UAS-(G3C2T)20*	This manuscript	N/A	Fig 1
*D. melanogaster: UAS-(G3C2T)33*	This manuscript	N/A	Fig 1
*D. melanogaster: UAS-(G3C2T)90*	This manuscript	N/A	Fig 1
*D. melanogaster: UAS-(GL-DPR)33*	This manuscript	N/A	Figs 3–5 and 7
*D. melanogaster: UAS-(AW-DPR)33*	This manuscript	N/A	Figs 3–5 and 7
*D. melanogaster: UAS-(PG-DPR)33*	This manuscript	N/A	Figs 3–7
*D. melanogaster: UAS-(AW-tandem-STOP)33*	This manuscript	N/A	Fig 6
*D. melanogaster: UAS-(AW-codon-mod)33*	This manuscript	N/A	Fig 6
*D. melanogaster: UAS-(PG-tandem-STOP)33*	This manuscript	N/A	Fig 6
*D. melanogaster: UAS-(PG-codon-mod)33*	This manuscript	N/A	Fig 6
*D. melanogaster: UAS-(AW-DPR)43-HFV*	This manuscript	N/A	Fig 3
*D. melanogaster: UAS-(PG-DPR)70-HFV*	This manuscript	N/A	Fig 3
*D. melanogaster: UAS-(PG-DPR)100*	This manuscript	N/A	Fig 3
*D. melanogaster: P{UAS-mCD8:GFP.L}2*	Bloomington Drosophila Stock Center	BDSC: 5137	Figs 1 and 4
*D. melanogaster: P{UAS-spt4*^*RNAi*^, *TRiP}*	Bloomington Drosophila Stock Center	BDSC: 31194	Fig 8
*D. melanogaster: P{UAS-spt4*^*RNAi*^, *KK}*	Vienna Drosophila Resource Center	VDRC: 108459; FlyBase: FBgn0028683	Fig 8
*D. melanogaster: P{GAL4 GMR}YH3/TM3, Sb*	Bloomington Drosophila Stock Center	BDSC: 84247	Fig 5
*D. melanogaster: P{GAL4-elav.L}2/CyO*	Bloomington Drosophila Stock Center	BDSC: 8765	Figs 3 and 5
*D. melanogaster: P{GAL4-OK371}*	Bloomington Drosophila Stock Center	BDSC: 26160	Figs 1, 4, 6 and 8
*D. melanogaster: P{GAL4-71B}*	Bloomington Drosophila Stock Center	BDSC: 1747	Fig 7
*D. melanogaster: P{GAL4-act5C}*	Bloomington Drosophila Stock Center	BDSC: 4414	Fig 4
*D. melanogaster: P{GAL4-repo}*	Bloomington Drosophila Stock Center	BDSC: 7415	Fig 4
*D. melanogaster:* w[1118]	Bloomington Drosophila Stock Center	BDSC: 3605	Figs 3–9

### Molecular cloning & transgenesis

All cloning steps were performed according to standard protocols. Restriction enzymes and DNA ligase were purchased from New England Biolabs (NEB), while oligonucleotides and gene fragments were purchased from Integrated DNA Technologies (IDT). All of these reagents were used following the manufacturer’s sheet. The oligonucleotide sequences were listed in Table 2.

**Table 2:** Oligonucleotides used in this study.

	Oligonucleotide sequence	Purpose
#1	CTAGCCATGGAGGTCTCAGGCCTGGGCCTGGGCCTGGGCCTGGGCCTGGTCTTCGAGCT	to clone (G3C2T)5
#2	CGAAGACCAGGCCCAGGCCCAGGCCCAGGCCCAGGCCTGAGACCTCCATGG	
#3	CTAGCAGATCTTAAATAAAATAAGGTCTCA	to clone non-ATG construct
#4	GGCCTGAGACCTTATTTTATTTAAGATCTG	
#5	GATCTACGCGTATGGAGGGTCTCA	to clone ATG to translate PG repeated peptide
#6	GGCCTGAGACCCTCCATACGCGTA	
#7	GATCTACGCGTATGGAGGTCTCA	to clone ATG to translate GL repeated peptide
#8	GGCCTGAGACCTCCATACGCGTA	
#9	GATCTACGCGTATGGAGCGGTCTCA	to clone ATG to translate AW repeated peptide
#10	GGCCTGAGACCGCTCCATACGCGTA	
#11	GGCCTGGGCCTGGGCCTGGGCCTGGGCCTGGGTAATAGG	to clone PG-tandem-STOP
#12	GGCCCCTATTACCCAGGCCCAGGCCCAGGCCCAGGCCCA	
#13	GGCCTGGGCCTGGCCCTGGACCTGGTCCAGGGCCAGGCCCAGGACCAGGTCCGGGGCCGGGCCCGGGACCGGGTCCCGGGCCCGGCCCCGGACCCG	to clone PG-codon-mod
#14	GGCCCGGGTCCGGGGCCGGGCCCGGGACCCGGTCCCGGGCCCGGCCCCGGACCTGGTCCTGGGCCTGGCCCTGGACCAGGTCCAGGGCCAGGCCCA	
#15	ATGGCCCTGGAGACGGTGCCGAAGGACCTG	*SUPT4H1*-forward primer for human fibroblast qPCR
#16	CTAGGTCTTTATAGCTGTGTCTCTGGATTTGTA	*SUPT4H1*-reverse primer for human fibroblast qPCR
#17	GACGCGATACCCAAGGATCTG	*Spt4*-forward primer for *Drosophila* qPCR
#18	TTTCACAGCCATCAGTCTCAAAT	*Spt4*-reverse primer for *Drosophila* qPCR
#19	CCATGGAGAAGGCTGGGG	*GAPDH*-forward primer for human fibroblast qPCR
#20	GGTCATGAGTCCCACGA	*GAPDH*-reverse primer for human fibroblast qPCR
#21	ACTTCATCCGCCACCAGTCG	RP49-forward primer for *Drosophila* qPCR
#22	CGGGTGCGCTTGTTCGATCC	RP49-reverse primer for *Drosophila* qPCR
	**DNA fragment**	
	CTCGAGTACCCATACGATGTTCCGGACTATGCGGGCTATCCCTACGACGTCCCGGACTATGCAGGATCCTATCCATACGACGTTCCAGATTACGCTCGGTACCGACTACAAAGACCATGACGGTGATTATAAAGATCATGACATCGATTACAAGGATGACGATGACAAGCCCCGGGGGCAAGCCCATCCCCAACCCCCTGCTGGGCCTGGATAGCACCTCTAGA	3xHA-3xFlag-V5 tag

To construct the SCA36 (G3C2T)n repeated sequence and the associated mutants, we employed the PCR-free direct cloning method adapted from Scior *et al* [20]. As depicted in the cloning strategy (S1 Fig), the BssHII-XhoI digested fragment from pUAST-attB [21] was first sub-cloned to pEGFP-N1 (Clontech) to obtain pEGFP-N1-pUASTattB(BX) to facilitate the following cloning process. The #1 and #2 oligonucleotides containing BsaI-5xG3C2T repeats-BbsI were annealed and then cloned into pEGFP-N1-pUASTattB(BX) via NheI and SacI to obtain pEGFP-N1-pUASTattB(BX)-(G3C2T)5. To extend the number of G3C2T repeats, the pEGFP-N1-pUASTattB(BX)-(G3C2T)5 was digested by BsaI-SacI to obtain the fragment as the insert. A parallel BbsI-SacI digestion of pEGFP-N1-pUASTattB(BX)-(G3C2T)5 generated the vector. Subsequently, the insert and vector ligation resulted in the extended G3C2T repeat numbers, e.g., pEGFP-N1-pUASTattB(BX)-(G3C2T)10. The iterative of this cloning process results in desired constructs with (G3C2T)n repeats in this study. To obtain the no-ATG constructs, the annealed #3 and #4 oligonucleotides were inserted into the designated pEGFP-N1-pUASTattB(BX)-(G3C2T)n (S1 Fig). To obtain ATG-containing constructs, annealed #5 and #6 (for translating Proline-Glycine (PG) repeated peptide), #7 and #8 (for translating Glycine-Leucine (GL) repeated peptide), and #9 and #10 (for translating Alanine-Tryptophan (AW) repeated peptide) were cloned into the designated pEGFP-N1-pUASTattB(BX)-(G3C2T)n. These constructs were confirmed by Sanger sequencing. The transgenic flies were used in Figs 3E, 3F and 4–6.

**Fig 1:**
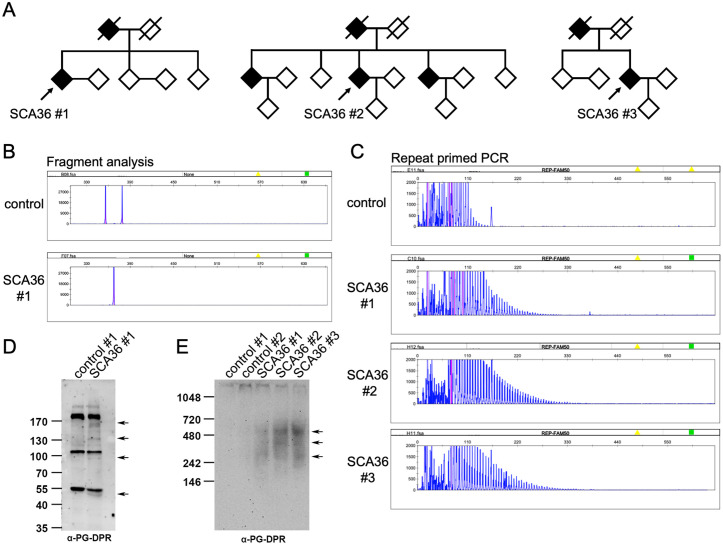
Genetic analysis of *NOP56* (GGCCTG)n repeat expansions. **(A)** Three unrelated pedigrees with SCA36 characterized in this study. Affected individuals are indicated by filled symbols, and index cases from individual families underwent genetic analysis and further skin biopsy are marked with arrows and labeled as SCA36_#1, SCA36_#2, and SCA36_#3, respectively. Deceased individuals are indicated by a diagonal slash through the symbol. **(B)** Fragment analysis using fluorescently labeled PCR primers targeting the *NOP56* repeat region. A control individual (*top*) shows two distinct peaks within the normal size range, indicating absence of repeat expansion. In contrast, the SCA36_#1 patient (*bottom*) shows a single allele within the normal range, with the expanded allele undetectable due to its large size, consistent with a heterozygous repeat expansion. **(C)** Repeat-primed PCR (RP-PCR) confirms the presence of pathogenic hexanucleotide (GGCCTG)n repeat expansions in the *NOP56* gene. While the control shows a regular stutter pattern terminating beyond the expected repeat region, all three SCA36 patients exhibit a characteristic sawtooth pattern with peak periodicity, consistent with an expanded repeat region and indicative of SCA36. **(D)** Immunoblot image from a denaturing SDS-PAGE gel showing the detection of DPR species by the anti-PG antibody in SCA36 patient samples. **(E)** Representative immunoblot image from a native (non-denaturing) gel showing detection of PG-DPR utilizing the anti-PG antibody.

**Fig 2:**
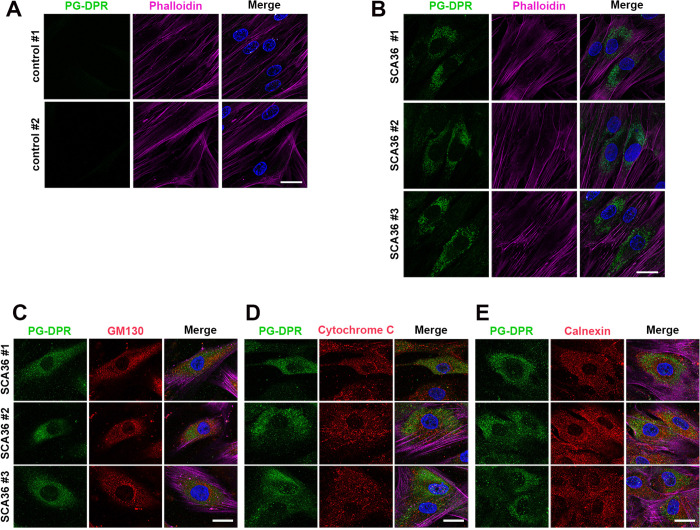
PG-DPR predominantly exhibits a diffuse distribution within the cytoplasm. Representative immunofluorescence of the fibroblast from healthy controls and SCA36 patients. **(A)** Absent PG-DPR signal in samples from two healthy individuals. **(B)** Diffused cytoplasmic distribution of the PG-DPR signals in the cells from three individual SCA36 patients. (Blue) DAPI staining to label nuclei; (Green) anti-PG immunofluorescence; (Magenta) Phalloidin staining for F-actin. Scale bar: 25 μm. (C-E) Co-immunostaining of PG-DPR with organelle-specific markers was performed to assess subcellular localization in SCA36 fibroblasts. **(C)** Golgi apparatus was labeled with GM130 (red). **(D)** Mitochondria was labeled with cytochrome C (red). **(E)** Endoplasmic reticulum with calnexin (red). PG-DPR showed partial perinuclear overlap with ER markers but remained predominantly cytoplasmic without specific subcellular colocalization. Scale bars: 10 μm.

**Fig 3:**
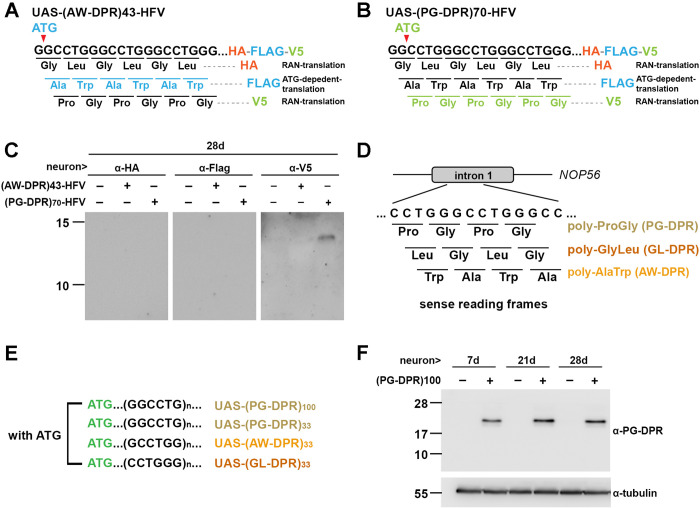
Detection of PG-DPR in adult fly brains. **(A, B)** Schematics of ATG-containing (G3C2T)43-HA-Flag-V5-tagged (HFV-tagged) and (G3C2T)70-HA-Flag-V5-tagged (HFV-tagged) constructs utilized to compare the effects of canonical and non-canonical translation. **(A)** The ATG codon is designed in-frame with Flag-tagged AW-DPR, while HA-tagged GL-PRD and V5-tagged PG-PRD might arise from non-ATG related translation. **(B)** The ATG codon is in-frame with V5-tagged PG-DPR. Whereas, HA-tagged GL-PRD and Flag-tagged AW-PRD might be produced through non-canonical initiation. **(C)** Western blots of fly head extracts expressing (G3C2T)70-HFV construct showing detectable level of V5-tagged PG-DPR. HA-tagged GL-DPR and Flag-tagged AW-DPR were below the detection threshold. n = 5 independent experiments; 15 heads per experiment. **(D)** Schematics of putative DPRs translated from three reading frames of the sense expanded (G3C2T)n at *NOP56* locus. **(E)** Schematic diagram of engineered expended (G3C2T)n constructs with ATG codons inserted into each of the three possible reading frames to drive frame-specific DPR translation. (F) Representative Western blot of PG-DPR detected by anti-PG antibody in elav-Gal4-driven fly head lysates expressing 100 repeats of PG-DPRs. Flies were aged to post-eclosion day 7, 21, and 28 to assess age-dependent DPR accumulation. n = 7 independent experiments; 15 heads per experiment.

**Fig 4:**
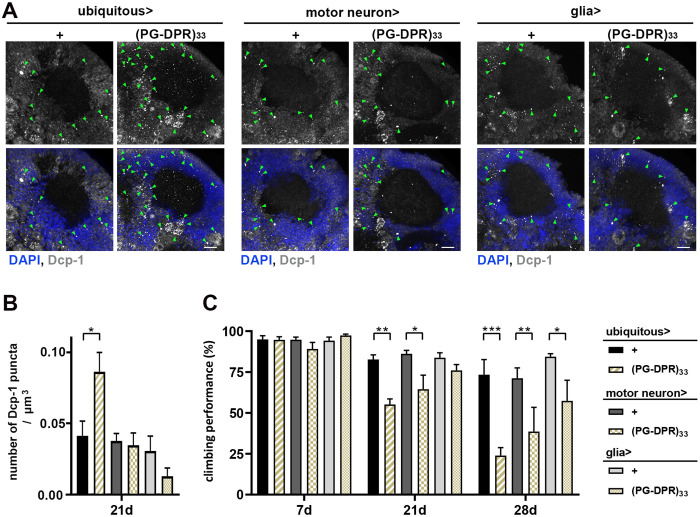
Cell-type related toxicity effect of PG-DPR. Transgenic expression with PG-DPR of 33 repeats (PG-DPR)33 driven by ubiquitous (act5C-Gal4), glutamatergic motor neuron-specific (OK371-Gal4), and glial-specific (repo-Gal4) drivers. **(A)** The adult fly brains were dissected to assess the effects of (PG-DPR)33. *Left panel*, prominent signals of stress-related apoptosis marker Dcp1 (green arrowhead) when ubiquitous expression of (PG-DPR)33. *Middle panel*, expressing PG-DPR in motor neuron display milder Dcp1-positive puncta. *Right panel*, glial expression exhibits less Dcp1 signals. **(B)** Statistical analysis of Dcp1 puncta showing significant increased Dcp1 signals when ubiquitous (PG-DPR)33 expression compared to neuron- or glia-specific expression (Data represent mean ± SEM; n = 6; *p < 0.05; one-way ANOVA Sidak’s multiple comparison test). **(C)** Negative geotaxis assay at 7, 21 and 28 days post-eclosion. Ubiquitous and motor neuron expression of (PG-DPR)33 demonstrated impaired climbing ability after 21 days post-eclosion. Glial expression of (PG-DPR)33 showed motor impairment at 28 days post-eclosion. (Data represent mean ± SEM; n = 3-4 independent experiments; 15-20 flies per experiment; *p < 0.05, **p < 0.01, ***p < 0.001; two-way ANOVA Tukey’s multiple comparisons test).

**Fig 5:**
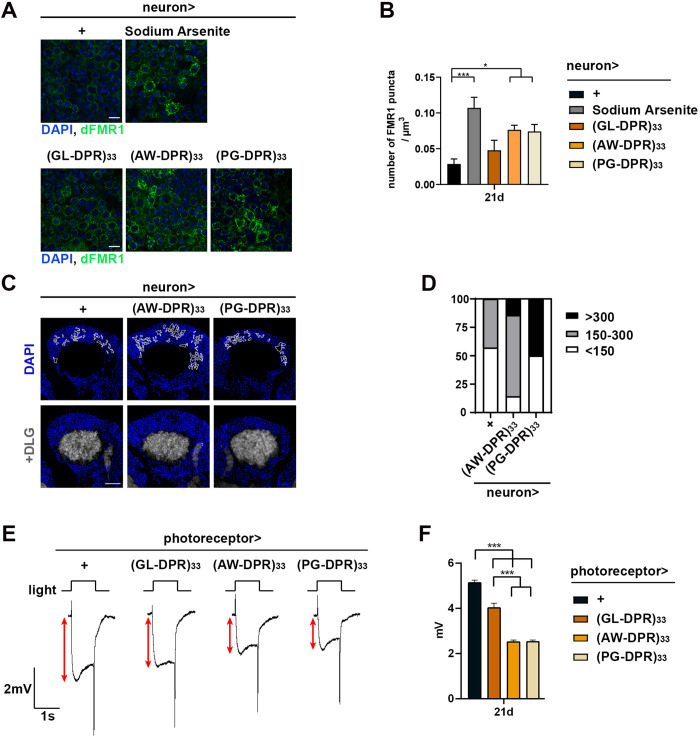
DPR-expressing flies exhibit stress granule formation, neurodegeneration loss and impaired neuronal activities. Transgenic expression of individual DPRs of 33 repeats in the fly brain and photoreceptor demonstrated the deteriorating effects from the DPRs. **(A)** Representative immunofluorescence images of adult fly brains expressing individual DPRs driven by the pan-neuronal elav-Gal4 driver at day 21 post-eclosion. Anti-dFMR1 antibody (green) targeting Drosophila fragile X mental retardation protein (FMRP) serves as a mark for stress granule formation, and nuclei are labeled with DAPI (blue). 1 mM sodium Arsenite-treated flies served as a positive control. Scale bar: 5 μm. n = 6-12 brains. **(B)** Quantification of FMRP signal intensity showed increased FMRP-labeled stress granule in Arsenite-treated, AW- and PG-DPR-expressing groups (Data represent means ± SEM; n = 6-8; *p < 0.05, ***p < 0.001; one-way ANOVA with Dunnett’s multiple comparisons test. **(C)** Detection of brain vacuoles (DAPI-sparing areas) at day 45 post-eclosion. Brains expressing PG-DPR showed the most extensive vacuolation, indicative of pronounced neuronal loss. Scale bar: 20 μm. **(D)** Quantitative analysis of vacuole burden categorized total vacuolar area into three severity levels: mild (<150 μm^2^), moderate (150-300 μm^2^), and severe (>300 μm^2^), respectively. **(E)** Representative electroretinogram (ERG) recordings from flies expressing GL-, AW-, or PG-DPRs under the GMR-Gal4 driver at day 21 post-eclosion. The red double arrow indicates receptor potential amplitude in response to 1-second white light stimulation. **(F)** Quantification of the receptor potentials from **(E)**. AW- and PG-DPR expression resulted in the prominent reduction of receptor potentials (Data represent means ± SEM; n = 3 independent experiments; 8-11 animals per experiment; *p < 0.05, **p < 0.01, ***p < 0.001; one-way ANOVA with multiple comparisons).

**Fig 6:**
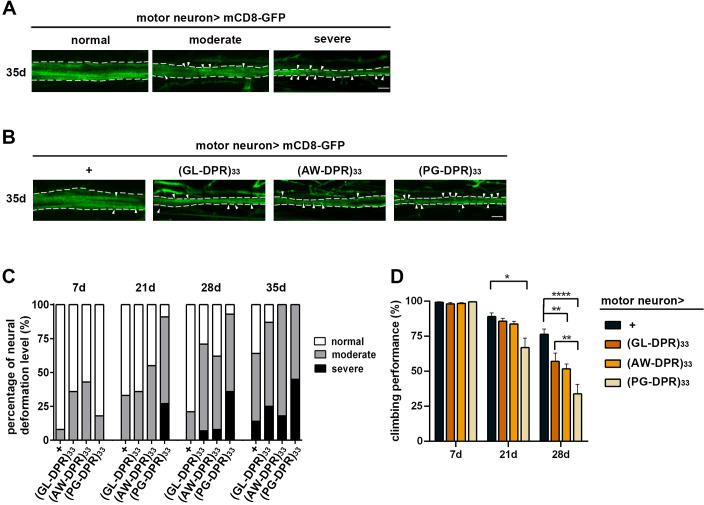
PG-DPR exhibits the most severe neurotoxicity in Drosophila motor neurons. To compare the relative toxicity of individual dipeptide repeats (DPRs), GL-, AW-, and PG-DPRs of equal repeat length were expressed in adult fly neurons using ATG-containing constructs aligned to their respective reading frames. **(A)** Motor axon was visualized using the membrane-anchored mCD8-GFP driven by the OK371-GAL4. Representative confocal images show normal structure (*left*), moderate deformation (*center*), and severe deformation (*right*). (Green) mCD8-GFP; arrowheads indicate bright puncta suggestive of protein aggregates in the *Drosophila* fore femur axons. **(B)** Representative confocal images of axon bundles from flies at post-eclosion day 35 expressing (DPRs)33. Scale bar: 10 μm. **(C)** Quantification of axon deformation of flies expressing individual DPRs at day 7, 21, 28, 35 post-eclosion (n = 5 independent experiments; 3-5 animals per experiment), showing age-dependent axon deformation. **(D)** Negative geotaxis analyses of DPRs-expressing flies demonstrated age-associated declines of climbing performance at day 21 and 28 post-eclosion, with most severe phenotype observed in flies expressing PG-DPR at day 28 (Data represent means ± SEM; n = 10-12 independent experiments; 15-20 animals per experiment; *p < 0.05, **p < 0.01, ***p < 0.001; two-way ANOVA with post hoc analysis).

**Fig 7:**
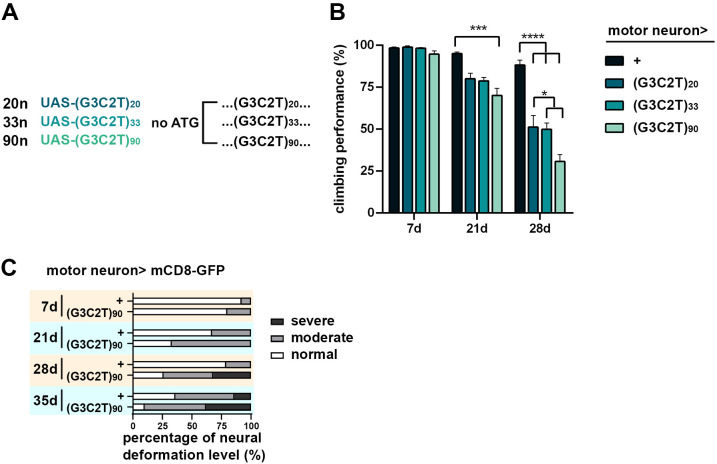
Repeat length- and age-related neurodegenerative effects of the expanded (G3C2T)n transcripts. **(A)** Schematics of ATG-excluding transgenic constructs of UAS-(G3C2T)n of various repeat lengths, namely 20n, 33n and 90n. **(B)** Negative geotaxis assay of flies with transgenic expression of 20n, 33n and 90n in motor neurons. Individual UAS-(G3C2T)n hexanucleotide repeats were expressed in glutamatergic motor neuron using OK371-GAL4 driver. Climbing performance was assessed at days 7, 21, and 28 post-eclosion (Data represent means ± SEM; n = 7 independent experiments; 20-25 animals per experiment; *p < 0.05, **p < 0.01, ***p < 0.001, ****p < 0.0001; two-way ANOVA with post hoc tests). **(C)** The classifications of neural morphology were defined as at Fig 6A. Quantification of axonal deformation in flies expressing (G3C2T)90 repeats (n = 7 independent experiments; 3-5 animals per experiment).

The obtained pEGFP-N1-pUASTattB(BX)-(G3C2T)n plasmids (non-ATG) were sub-cloned to pUAST-attB via BssHII-XhoI sites for further transgenic fly generation in Figs 7 and 9A–9C. To monitor the potential peptide products from (G3C2T)n repeats, we cloned a 3xHA-3xFlag-V5 tag (from a synthetic gene fragment) into pUAST-attB to construct pUAST-attB-HFV. Each tag is in a different reading frame to allow the identification of all possibilities of peptide synthesis of the repeated sequence, a concept similar to the previous report [22]. This pUAST-attB-HFV plasmid was used as a scaffold to accommodate the (G3C2T)n repeats from pEGFP-N1-pUASTattB(BX)-(G3C2T)n through the sub-cloning via BssHII-XhoI sites. The resulting plasmids were used to generate transgenic flies. The transgenic flies were used in Fig 3A–3C.

**Fig 8:**
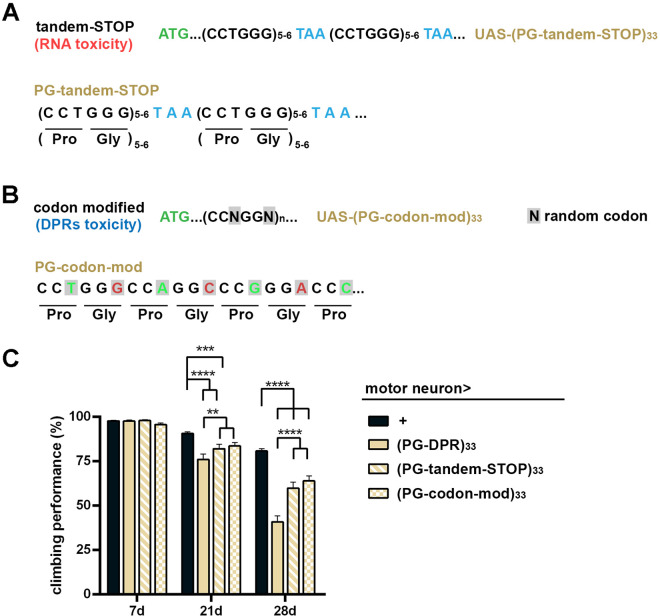
Combinatorial neurodegenerative effects from DPR and (G3C2T)n transcripts in SCA36 flies. **(A)** Schematics of PG-tandem-STOP construct designed to disrupt PG-DPR production. Stop codons were inserted every 5-6 dipeptides to reduce the formation of extended PG-DPR. **(B)** Schematics of PG-codon-modified construct. The third nucleotide of each codon was modified to disrupt the repetition at DNA/RNA level but reserve the amino acid repetition of PG-DPR. **(C)** Climbing assay to evaluate locomotor function in adult flies expressing PG-DPR, PG-tandem-STOP, or PG-codon-modified constructs under the motor neuron-specific OK371-GAL4 driver. Behavioral assessments were performed at 7, 21, 28 days post-eclosion (Data represent means ± SEM; n = 7 independent experiments; 15-20 animals per experiment; *p < 0.05, **p < 0.01, ***p < 0.001, ****p < 0.0001; two-way ANOVA with post hoc comparison).

The PG-tandem-STOP constructs were generated via the same strategy above, except the initial oligonucleotides are #11-#12. Similarly, the random codon modification constructs PG-codon-mod) were generated by the oligonucleotides #13-#14. The transgenic flies were used in Fig 8.

### Immunohistochemistry for *Drosophila*

Adult fly brains were dissected at post-eclosion day 21 following immersion in 1x PBS. The dissected brains were fixed in 4% PFA for 20 minutes at room temperature, then washed three times with 1% PBST (PBS containing 0.1% Triton X-100) for 10 minutes each. Brains were subsequently blocked in 1% BSA in 0.25% PBST for immunostaining. Primary antibodies used included mouse anti-dFMR1 (1:100; DSHB, AB_528252) and rabbit anti-Dcp-1 (1:100; Cell Signaling Technology, #9578), followed by appropriate secondary antibodies. Samples were mounted in Vectashield mounting medium (Vector Laboratories) and imaged using a Leica SP8 confocal microscope operated with LAS AF software. For quantification, the number of puncta was analyzed using the Spot detection function within Imaris software. The volume of each brain region was determined using the Surface rendering function. Puncta density was calculated as the number of puncta per unit volume (puncta count/volume). The antibodies used in this study are listed in the Table 3.

**Table 3:** Antibodies used in this study.

Antibody	Source	Identifier	Concentration
Mouse anti-*Drosophila* discs large	Developmental Studies Hybridoma Bank (DSHB)	4F3; RRID: AB_178203	1:200
AffiniPure Rabbit Anti-Horseradish Peroxidase	Jackson ImmunoResearch	RRID: AB_2314648	1:200
Mouse anti-poly GP	Developmental Studies Hybridoma Bank (DSHB)	AB_2753318	1:200 (microscopy image); 1:500 (western blot)
Rabbit anti-ATP5A	Abcam	ab14748	1:100
Rabbit anti-calnexin	Santa Cruz	sc-11397	1:200
Rabbit anti-cytochrome C	Abcam	EPR1327	1:200
Rabbit anti-GM130	Abcam	ab30637	1:100
Rabbit anti-Dcp-1	Cell Signaling Technology	Cat# 9578	1:100
Mouse anti-dFMR1,5A11	DSHB	Catalog #5A11	1:100
Mouse anti-alpha-tubulin	Developmental Studies Hybridoma Bank (DSHB)	AB_1157911	1:10000
Alexa 568-conjugated phalloidin	Thermo Fisher	Cat# 12,380	1:200
Alexa 647-conjugated phalloidin	Thermo Fisher	Cat# 8940S	1:200
Rabbit anti-FMRP	Abcam	ab17722	1:200
Rabbit Anti-GRP78 (BiP)	StressMarq	Cat# SPC-180; RRID: AB_10804549	1:200
Rabbit anti-ref(2)p (p62)	Abcam	ab178440	1:200
anti-rabbit-568	Invitrogen	catalog #A11004, RRID: AB_141371	1:100
Cy3 anti-rabbit IgG	Jackson ImmunoResearch,	Cat# 111-165-003; RRID: AB_2338000	1:500
anti-mouse-647	Jackson ImmunoResearch		1:500

### Immunofluorescence analyses for fibroblast

Fibroblast-attached coverslips were rinsed twice in 1x PBS (containing 136 mM NaCl, 2.5 hsiaoctmM KCl, 1.5 mM KH2PO4, and 6.5 mM Na2HPO4, pH 7.4), and then fixed with methano on ice for 20 minutes. Fibroblasts were blocked and permeabilized with blocking buffer containing 0.1% (v/v) Triton X-100, 5% normal goat serum, 20 mM phosphate buffer, and 0.45 M NaCl, pH 7.4, for 60 minutes at 4°C. The fibroblasts were then incubated with primary antibodies included a mouse anti-poly-PG primary antibody (1:50; Developmental Studies Hybridoma Bank, Iowa City, IA, USA), rabbit anti-ATP5A (1:100; Abcam, Catalog #ab14748, Cambridge, UK), rabbit anti-calnexin (1:200; Santa Cruz, Catalog #sc-11397, TX, USA), rabbit anti-GM130 (1:100; Abcam, Catalog #ab30637), in blocking buffer overnight at 4°C. Next, fibroblasts were washed three times in blocking buffer at room temperature. Immunoreactivity was visualized with a goat anti-mouse secondary antibody conjugated to Alexa Fluor 488 (1:100; Invitrogen catalog #A11001, RRID:AB_2534069), and goat anti-rabbit secondary antibody conjugated to Alexa Fluor 568 (1:100; Invitrogen catalog #A11004, RRID:AB_141371). Fibroblasts were then washed three times in 1x PBS.

### Microscopy

Images of the fly tissues including larval salivary gland, leg axon, and brain were captured with Zeiss LSM880 confocal laser scanning microscope (Zeiss microscopy, Germany). Neuromuscular junction (NMJ) and salivary gland samples were analyzed on Zeiss ApoTome.2. The human fibroblasts were imaged through Leica TCS SP8 LIGHTNING confocal microscope (Leica Camera, Germany).

### Climbing assay

The climbing assay was adapted from Hung *et al* [23]. Briefly, locomotor activity was measured by counting the number of flies that climbed 7 cm in 3 seconds. Groups of 20 flies were placed in connected vials and observed for 1 minute, with 50 seconds of rest between trials. The entire process was video recorded for analysis.

### Electroretinogram (ERG)

ERGs were performed as described previously by [24]. Briefly, flies were immobilized on glass slides, and ERGs were recorded during one-second light-on and light-off cycles using electrodes filled with 2 M NaCl. Each genotype and experimental condition were tested in triplicate, with at least 10 recordings per replicate.

### Human fibroblasts

Written informed consents were provided by the participants in accordance with relevant regulatory guidelines. The study was approved by the Institutional Review Board of Taipei Veterans General Hospital. The information regarding skin biopsy acquisition were listed in Tables 4 and 5. A three-millimeter punch skin biopsy was obtained from the patients by an authorized specialist. The tissues were then cut into several pieces, and the standard sample was examined by routine histopathology studies for clinical purposes. A small part of residue tissue was rinsed by 1x PBS for three times. The harvested tissue was shredded by using a dissection scissor in the culture dish. The tissue was then covered by coverslips in 20 ml DMEM culture medium supplemented with 10% fetal bovine serum (Hyclone, South Longan, UT, USA), 1 mM sodium pyruvate, 100 units/ml penicillin, and 50 μg/ml streptomycin at 37°C in humidified incubator with 5% CO2 and 95% air. About 20 days after the procedure, fibroblasts grown from the tissue were dissociated with 0.25% trypsin solution. The isolated fibroblasts were seeded into new culture dishes for maintenance and further experiments. The human fibroblast cells were cultured in DMEM medium (Catalog #11965–084, GIBCO,Gaithersburg, MD, USA) supplemented with 10% fetal bovine serum (Hyclone, South Longan, UT, USA) and maintained at 37°C in a 5% CO_2_ humidified incubator. The cells were passaged when reaching confluency.

**Table 4:** The clinical information of the SCA36 patients provided fibroblast cells for the experiments in this study.

	SCA36_#1	SCA36_#2	SCA36_#3
Gender	F	M	F
Age at onset	50	46	45
Age at examination	57	48	49
Truncal ataxia	++	++	++
Limb ataxia	+	++	++
Saccadic pursuit	+	+	+
Ataxic dysarthria	+	+	+
Dysphagia	+	–	–
Tongue atrophy or fasciculation	–	Mild	Mild
Muscle atrophy and fasciculation	+	–	–
Hyperreflexia	+++	–	–
Hearing impairment	–	+	–
Impaired vibration	+	–	–
Other symptoms	Tremors	Nystagmus	Blurred vision

**Table 5:** Fibroblast cell lines used in this study.

Identifier	Sex of the individual	Age at the sample obtained	Source
Control_#1	Female	46	Taipei Veterans General Hospital
Control_#2	Female	55	Taipei Veterans General Hospital
SCA36_#1	Female	57	Taipei Veterans General Hospital
SCA36_#2	Male	50	Taipei Veterans General Hospital
SCA36_#3	Female	49	Taipei Veterans General Hospital

### Western blotting

Western blotting was performed as previously described [25]. Protein extracted from fly heads or human fibroblast cell lines, sample lysate from tissue protein extraction reagent (Catalog #78510, Thermo Fisher Scientific, USA), and cell lysis buffer containing the following (in mM): 150 NaCl, 5 EDTA, and 50 Tris-HCl, pH 7.6, 1% Triton X-100, 1mM PMSF, 1mM DTT and containing a protease inhibitor mixture. Protein was separated by SDS-PAGE and transferred to polyvinylidene difluoride membranes (PVDF, Millipore) as per the manufacturer’s instructions (Bio-Rad, USA). PVDF membranes were incubated with 5% skimmed milk in TBST (10 mM Tris (pH 8.0), 150 mM NaCl, and 0.1% Tween 20) for 1 hour at room temperature, following washed with TBST for 10 minutes for 3 times, and incubated with primary antibodies at 4°C overnight.

Native polyacrylamide gel electrophoresis (NativePAGE Novex Bis-Tris Gel System, Invitrogen) was performed according to the manufacturer’s instructions. Following electrophoresis, proteins were electrotransferred onto PVDF membranes using the XCell II Blot Module (Invitrogen) and NuPAGE Transfer Buffer to preserve the near-neutral pH conditions of the gel system. PVDF membranes were pre-activated with methanol prior to assembly. After transfer, membranes were briefly fixed in acetic acid, rinsed, and air-dried. For immunodetection, membranes were rehydrated with methanol, washed in deionized water, and processed by standard Western blotting procedures, including blocking, antibody incubation, and chemiluminescent detection. Molecular weight estimation was performed using the NativeMark Unstained Protein Standard (Invitrogen, catalog: LC0725).

Detected using rabbit anti-Tubulin (1:5000; Abcam, Cambridge, UK), rabbit anti-Flag (1:5000; Sigma, St. Louis, MO, USA), rat anti-HA (1:5000; Roche, Basel, Switzerland), mouse anti-PG-DPR (1:500; Developmental Studies Hybridoma Bank (DSHB), Iowa City, IA, USA), or mouse anti-V5 (1:1000; Abcam, Cambridge, UK), antibodies. Membranes were washed three times with TBST for 10 minutes before incubation with secondary antibodies in

TBST for 1 hour at RT. Blots were exposed to the secondary antibody which were horseradish-per-oxidase-conjugated goat anti-mouse IgG (1:5000; Jackson ImmunoResearch, West Grove, PA, USA), goat anti-rabbit IgG (1:5000; Jackson ImmunoResearch), or goat anti-rat IgG (1:5000; Santa Cruz, Dallas, TX, USA), and revealed by an enhanced chemiluminescence detection system (Thermo Fisher Scientific). Blots were then washed with TBST for three times and revealed by an enhanced chemiluminescence detection system (PerkinElmer, NEL120E001EA; GeneTex, GTX14698; Thermo Scientific, 34580), and captured by the BioSpectrum 600 Imaging System (UVP Ltd). Quantification of western blotting results was performed using ImageJ software (RRID:SCR_003070).

### Quantitative PCR (qPCR) for human fibroblasts and *Drosophila*

Total RNA was extracted from human fibroblast cell lines using TRIzol reagent (Sigma), followed by phenol/chloroform separation. RNA was reverse transcribed into cDNA using the High-Capacity cDNA Reverse Transcription Kit with RNase Inhibitor (Thermo Fisher). Reverse transcription was performed with incubation at 25 °C for 10 min and 37 °C for 120 min, followed by heating at 85 °C for 5 min. For qPCR analysis of human fibroblast samples, the resulting cDNA was diluted with nuclease-free water and amplified in 25 µL reactions using SYBR Green PCR Master Mix (Thermo Fisher) with 500 nM forward and reverse primers. Primer sequences are provided in Table 2.

For Drosophila samples, adult flies were homogenized, and RNA was isolated using the Quick-RNA Miniprep Kit (Zymo Research, Cat# R1055). RNA concentrations were determined, and samples were normalized before cDNA synthesis. Reverse transcription was performed using IQ^2^ MMLV RT-Script (Bio-Genesis, Cat# BB-DBU-RT-001) with a cDNA Accessory kit(Bio-Genesis, Cat# MG-DR01611–1). qRT-PCR was conducted on a QuantStudio 5 Real-Time PCR System (Applied Biosystems) using the KAPA SYBR FAST qPCR Master Mix (2X)ROX Low Kit (KAPA Biosystems, Cat# KK4619). Primer sequences are listed in Table 2.

### Quantification and statistical analysis

Quantitative data were analyzed using appropriate statistical tests, including two-tailed unpaired Student’s t-tests, one-way ANOVA with Tukey’s multiple comparisons test, or two-way ANOVA with Bonferroni posttests. Graphs were generated using GraphPad Prism 5. Data in bar graphs are presented as mean ± SEM. Statistical significance was determined using a p-value threshold of 0.05. All statistical details are provided in the figure legends.

### AI tool usage

AI language tools, including ChatGPT and Gemini, were used to assist in the writing and editing of this manuscript. However, all data, analyses, and interpretations were conducted by the authors. Background-removed images used in the graphic abstract were generated using ChatGPT to enhance visual clarity and consistency. The conceptual design, layout, and overall composition of the graphic abstract were originally created by the authors.

## Results

### Characterization and neurotoxicity of dipeptide repeat proteins (DPRs) in cells from SCA36 patients

To investigate the molecular mechanism of SCA36, we conducted multimodal experiments including the fibroblasts we collected from SCA36 patients, the fly transgenic model, and the exogenous expression system. Fibroblast samples were obtained from three unrelated SCA36 patients, all presenting with late-onset cerebellar ataxia and variable degrees of motor system degeneration (Table 4). The diagnosis of SCA36 was confirmed by identifying *NOP56* hexanucleotide repeat expansions using repeat-primed PCR (Fig 1A–1C). Previous literature showed that the (G4C2)n expansion of *C9orf72* FTD/ALS can undergo either repeat-associated non-ATG (RAN) translation or canonical translation to produce PG-DPR. Likewise, these SCA36-related expanded (G3C2T)n repeats are likely to produce dipeptide repeat proteins (DPRs), including poly-PG, which were detected by immunoblotting with an anti-PG antibody (Fig 1D). The molecular weights of the PG-positive protein species ranged from approximately 250–500 kDa, corresponding to an estimated (G3C2T)n repeat length of around 1,135–2,272 hexanucleotide units (Fig 1E). These findings are consistent with previous reports demonstrating predominant PG-DPR expression in (G3C2T)n-transfected HEK293T cells, transgenic (G3C2T)n-expressing mice, and postmortem brain tissues from SCA36 patients [16,17]. The finding may support the occurrence of RAN translation or upstream ATG-related translation may contribute to this SCA36 (G3C2T)n-related PG-DPR [16,17,26]. To assess the subcellular localization of PG-DPR, we performed immunostaining on patient-derived fibroblasts. Immunofluorescence analysis revealed a absence of PG-DPR signal in the control cells, while diffuse cytoplasmic distribution of PG-DPR was prominent in the patient cells (Fig 2A and 2B). This PG-DPR signals exhibit small puncta with partial perinuclear overlap with endoplasmic reticulum (ER) markers but remained predominantly cytoplasmic without specific subcellular colocalization to the Golgi apparatus, or mitochondria (Fig 2C–2E). This localization pattern is consistent with previous findings [17], suggesting that PG-DPR exists as a soluble, non-aggregating dipeptide repeat protein.

### DPR exerts cell-type related toxicity in neuronal and non-neuronal tissues of *Drosophila*

To establish a SCA36 model in *Drosophila*, we generated a series of transgenic flies harboring the expanded sequences, wherein the expression of the repeats is controlled by the GAL4/UAS system [27]. To examine the toxicity caused by DPR in flies, 2 sets of transgenic flies were generated. In the first set, we inserted a tandem HA-Flag-V5 tag to the C-terminus of the (G3C2T)n expansion (Fig 3A and 3B). At the 5’ end, ATG was inserted in frame with the translation of AW or PG dipeptide. This design led to in-frame translation of each DPR frame with different epitope tag, resulting in the potential production of AW-DPR-FLAG and PG-DPR-V5, respectively. The transgenes were driven by a pan-neuronal driver elav-GAL4, and subsequent Western blotting was conducted to validate the tagged DPR in fly neuron. We detected the V5-tagged PG-DPR, whereas the FLAG-tagged AW-DPR was not detectable (Fig 3C). Our observation may suggest the expression level of the FLAG-tagged (AW-DPR)43- protein may be below the detection threshold or the predicted molecular weight of this protein is less than 10 kDa, which is likely too small to be efficiently resolved or retained using our current Tris-glycine-based western blot system. The second set was designed with an ATG start codon inserted in frame with each of the sense reading frames of (G3C2T)n, resulting in the possible translation of GL-, AW-, and PG-DPR, respectively (Fig 3D and 3E). However, Western blotting of the fly lysates only revealed an accumulation of PG-DPR in the aging fly (Fig 3F). These findings indicate the robust stability of PG-DPR, potentially suggesting its association with neurotoxic effects.

Furthermore, we expressed PG-DPR of 33 repeats (PG-DPR)33 transgenically using ubiquitous (act5C-Gal4), glutamatergic motor neuron-specific (OK371-Gal4), and glial-specific (repo-Gal4) drivers to assess the effects of PG-DPR in adult fly brains. Notably, when ubiquitously expressed via act5C-Gal4, we observed an increased signal of Dcp1-immunoreactive puncta, indicating the activation of stress-related apoptotic pathways. The Dcp1 signals were not prominent when expressed (PG-DPR)33 by motor neuron- and glial-specific drivers (Fig 4A and 4B). Intriguingly, negative geotaxis assays on these (PG-DPR)33 transgenic flies revealed locomotor defects most pronounced in flies with ubiquitous (PG-DPR)33 expression, followed by motor neuron-specific, and then glial-specific expression (Fig 4C). While glial expression also resulted in mild locomotor impairment at later stage, the effects were less severe than in neurons, suggesting that non-neuronal cells contribute to PG-DRP-induced toxicity, although to a lesser extent.

### Transgenic expression of SCA36-relevant DPRs exhibits stress granules in the fly brain and neurodegeneration in the motor and sensory neurons

SCA36 is considered a late-onset neurodegenerative disorder, typically affecting the central nervous system in adulthood [1–3]. Therefore, we used the adult fly brain as a test tube to investigate potential abnormalities upon individual DPRs expression. While PG-DPR has been shown to impair stress granule dynamics in a mammalian *C9orf72* model [28], no signs of stress granule assembly was observed in SCA36-related poly-PG-expressing cells [17]. The mixed results have prompted us to investigate the cellular impacts of each of the DPR. We stained the DPR-expressing tissue with FMRP (fragile X mental retardation protein) as markers for stress granules [29]. As a positive control, NaAsO_2_-treated flies exhibited robust FMRP immunofluorescence signals under confocal imaging, indicating the formation of stress granules. Similarly, brains expressing PG-, and AW-DPR showed less but significant accumulation FMRP signal, suggesting the presence of stress granule formation under basal conditions (Fig 5A and 5B). The vacuolization of the CNS resulting from neuronal loss was visualized by counterstaining of DAPI. PG-DPR-expressing flies exhibited significant vacuolation compared with wild-type controls at day 45 post-eclosing, indicating the degenerating brain (Fig 5C and 5D).

Sensorineural impairment has been observed in SCA36 patients [2,3]. Therefore, we asked whether expressing DPRs may also lead to functional deterioration in fly sensory system. To achieve this goal, individual DPRs were expressed specifically in the fly photoreceptor sensory neurons using the GMR-Gal4 driver. The photoreceptor function was assessed with electroretinogram (ERG). The amplitude of receptor potential serves as a readout for photoreceptor health, as the decrease in amplitude indicates neurodegeneration. The PG-DPR and AW-DPR groups exhibited a greater reduction in receptor potential compared to the GL-DPR group at day 21, suggesting the former two groups having a more pronounced toxic effect on the aging photoreceptors (Fig 5E and 5F). Taken together, our fly model demonstrated the significant involvement of DPRs-related toxicity in the pathogenesis of SCA36, with PG-DPR exhibiting the most notable neurotoxicity in the CNS as well as the sensory neurons.

Given motor neuron degeneration has been observed in patients with SCA36, we then investigated the neurotoxic effects of the DPR on the motor nerve bundle. To examine the impact on neural morphology, we marked the repeats-expressing neurons by co-expressing mCD8-GFP, a membrane-anchoring fluorescent protein, and examined the neural morphology with confocal microscopy. The morphological defect was classified from normal to severe based on bundle thickness and abnormal aggregations (Fig 6A) [30]. Transgenic DPR expression in motor neurons led to age-dependent degeneration, as revealed by noticeable neural deformation and aggregation within the axon bundle (Fig 6B). Among the three DPRs, PG-DPR exerted the most pronounced toxicity, evident from severe axon deformities compared to the other two DPRs upon aging (Fig 6C). Similarly, flies expressing PG-DPR in motor neurons showed a more rapid deterioration in locomotor activity when compared to the other two DPRs, indicating functional impairments in SCA36 fly models might be predominantly associated with PG-DPR (Fig 6D).

### Dissecting the relative contributions of HRR and DPR to SCA36 neurotoxicity

Previous studies in cultured Neuro2A cells and patient-derived iPSCs indicated that HRR of the expanded (G3C2T)n are the primary cause of neurotoxicity in SCA36 [11,12]. A *Drosophila* model of FTD/ALS demonstrated that the neurotoxicity in *C9orf72* correlates with the number of the expanded (G4C2)n repeats [31]. To examine the correlation between length of the repeated (G3C2T)n hexanucleotide and SCA36 neurotoxicity, our transgenic UAS flies was designed to carry the expanded (G3C2T)n of varying repeat lengths (hereafter referred to as 90n, 33n, and 20n, respectively) (Fig 7A). Since this set of constructs does not contain a start codon, the potential toxicity is attributed primarily to RNA transcripts of (G3C2T)n. To examine the neurotoxic effect, we used OK371-GAL4 to specifically express the transgenes in motor neurons, followed by evaluating the neural function. Negative geotaxis serves as a behavioral assay that reflects the locomotor activity of adult flies, potentially linked to their motor function. The HRR-expressing flies exhibited normal climbing activity at day 7 post-eclosion but showed a locomotor deficit at day 21 when compared to the wild-type controls, indicating adult-onset degeneration (Fig 7B). Notably, the deficits correlated with repeat length and age, with the 90n flies displaying more severe defects in climbing compared to those with shorter repeats at day 21 and 28 (Fig 7B). Then we examine the impact of HRRs on neural morphology by confocal imaging the neural morphology. 90n flies exhibited evident deformation of the axon bundle, and the deformation became more prominent at day 35 (Fig 7C), indicating an age-dependent morphological degeneration. These findings indicate a length- and age-dependent correlation between the (G3C2T)n HRR and the degree of neurodegeneration. This length-dependent toxicity observed in the SCA36 fly model may be in parallel with clinical observations in patients, where individuals with relatively shorter expansions within the pathogenic range tend to exhibit milder phenotypes [4].

While our findings and others suggest that neurotoxicity in SCA36 patients can be attributed to DPR, the impact of HRR may also exert additional impact on SCA36 pathogenesis as demonstrated in Fig 7 and observed in previous studies [11,12]. To pinpoint the primary cause of neurotoxicity, we engineered a set of constructs to distinguish between toxicity arising from HRR and that stemming from DPR (Fig 8A and 8B). The PG-tandem-STOP construct featured a stop codon inserted in every 5–6 dipeptide repeats, effectively disrupting the formation of extended DPR (Fig 8A). Conversely, the PG-codon-modified construct altered the third amino acid of each codon, thereby disrupting the repetitive nature of HRR while preserving the repetition of the DPR (Fig 8B). Expression of either the tandem-STOP or codon-modified transgenes in neurons caused locomotor decline, suggesting that both HRR and DPR contribute to the functional impairment in SCA36 (Fig 8C). Notably, while both transgenes caused significant locomotor decline, the deterioration was not as severe as the PG-DPR transgene alone which produces both HRR and DPR concomitantly (Fig 8C). Taken together, our findings demonstrate a combinatorial toxicity involving HRR and DPR underlying the pathogenesis of SCA36.

### Suppression of *Spt4* fails to ameliorate SCA36-related neurodegeneration

Finally, we interrogated whether the transcription elongation machinery, a potential therapeutic strategy for repeat expansion disorders, would ameliorate SCA36 neurotoxicity. The DRB (5,6-dichloro-1-β-d-ribofuranosylbenzimidazole) sensitivity-inducing factor (DSIF) complex, composed of the Spt4 and Spt5 subunits, plays a critical role in RNA elongation by stabilizing the RNA polymerase II (Pol II) elongation complex. These elongation factors may contribute the transcription of expanded repeat sequences into aberrantly elongated transcripts. Attenuating the function of Spt4 and Spt5 could therefore mitigate the production of pathogenic HRR [13,14]. Previous literatures have demonstrated in patient-derived cells from individuals with *C9orf72* mutation associated ALS/FTD, knockdown of the human ortholog of *Spt4* transcriptional elongation factor *SUPT4H1* significantly reduced the formation of RNA foci and suppressed the expression of repeat-associated non-AUG (RAN) translation proteins [14]. Other studies have also demonstrated that silencing the murine homolog Supt4h in mouse models of Huntington’s disease led to reduced expression of mutant huntingtin mRNA, decreased formation of polyQ aggregates, and improvement in disease-related phenotypes [32].

**Fig 9:**
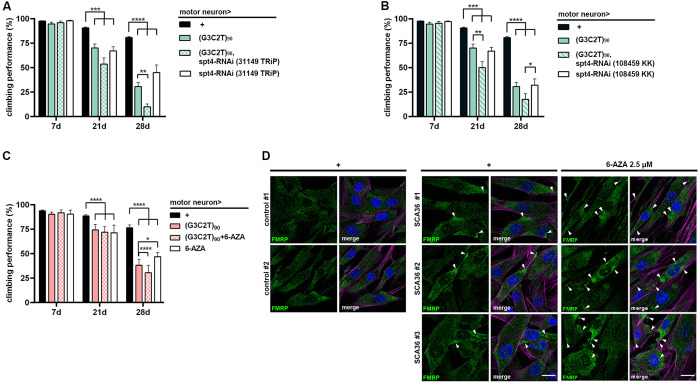
*Spt4* knockdown and 6-azauridine treatment exacerbate locomotor deficits and stress responses in SCA36 models. **(A, B)** Two independent *Spt4*-RNAi lines were used to knock down *Spt4* expression in glutamatergic motor neurons using OK371-GAL4. Climbing performance was assessed in adult flies at post-eclosion day 7, 21, and 28. Co-expression of *Spt4*-RNAi with the expanded (G3C2T)90 repeat led to significantly worsened locomotor deficits, especially at day 21 and 28, compared to flies expressing (G3C2T)90 alone, *Spt4*-RNAi alone, or controls. **(C)** 2.5 μM 6-Azauridine (6-AZA), an RNA synthesis inhibitor, was administered from the larval stage through adulthood. Negative geotaxis assays revealed that 6-AZA treatment further impaired locomotor performance in (G3C2T)90-expressing flies across all examined time points (day 7, 21, and 28) (Data represent means ± SEM; n = 7-9 independent experiments, 15-20 animals per experiment for *Drosophila* assays; n = 3 independent experiments for fibroblast studies; *p < 0.05, **p < 0.01, ***p < 0.001, ****p < 0.0001; two-way ANOVA). **(D)** Fibroblasts derived from three independent SCA36 patients were treated with 6-AZA and stained for FMRP (green) to assess stress granule formation. Aggregated FMRP-positive puncta were observed by confocal microscopy, with arrowheads indicating stress granules. Nuclei were counterstained with DAPI (blue). Scale bar: 15 μm.

To understand whether perturbation of transcription elongation machinery may contribute to the pathogenesis of SCA36, we firstly performed q-PRR to examine the expression profiles of *SUPT4H1* in patient-derived fibroblasts. Our results demonstrated a comparable expression patten of *SUPT4H1* in the health controls and SCA36 patient samples as at their basal expression level (S3A Fig). Although knockdown of *Spt4* leading to a reduction in RNA foci has shown beneficial effects in cellular models [12], its therapeutic effect has never been tested in a living organism. To address this, we assessed the functional impact of regulating *Spt4* in our fly SCA36 model and patient-derived fibroblasts. Two independent RNAi strains targeting *Spt4* were tested. The *Spt4* knockdown efficiency was verified by qPCR (S3B Fig). Surprisingly, knockdown of *Spt4* in the motor neuron using two independent RNAi strains was not able to ameliorate the locomotor defects of (G3C2T)n-induced neurotoxicity. Instead, it paradoxically exacerbated the phenotypic severity (Fig 9A and 9B). Similarly, treatment with 6-azauridine (6-AZA), a small molecule that potentially targets the Spt4/5 interaction to suppress the elongation machinery, also aggravated these defects (Fig 9C). Furthermore, 6-AZA treatment to the SCA36 fibroblasts induced stress granule accumulation in a dose-dependent manner (Fig 9D). These findings suggest that suppression of *Spt4*-rlated RNA elongation may not alleviate the locomotor defect of SCA36 flies, indicating that (G3C2T)n-driven neurodegeneration may proceed via pathways beyond the *Spt4*-dependent mechanisms.

## Discussion

**Fig 10:**
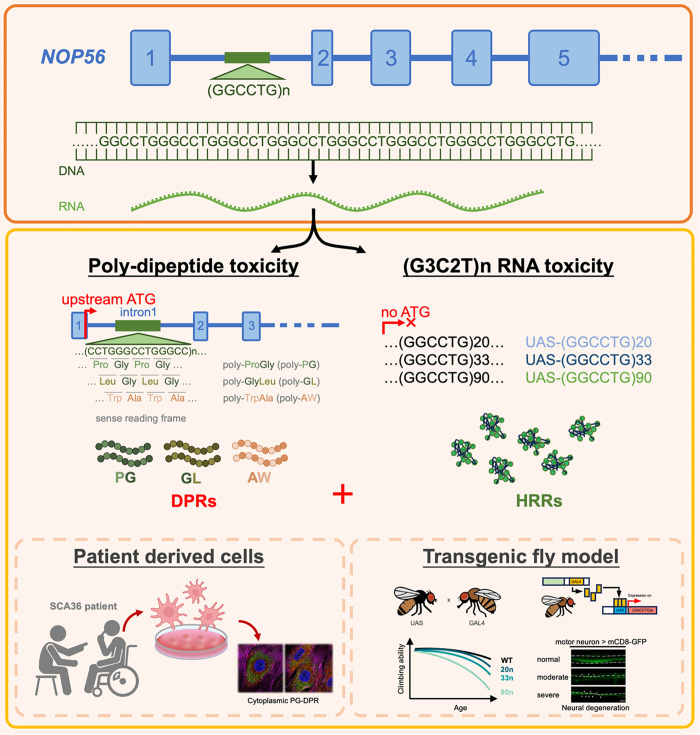
Combinatory toxic mechanisms of SCA36: DPR and HRR toxicities. Graphic abstract illustrates the pathogenic mechanisms of SCA36, caused by the (GGCCTG)n hexanucleotide repeat expansion in the first intron of *NOP56*. Expanded repeats may be transcribed into RNA and translated into dipeptide repeats, giving rise to two synergistic toxic mechanisms: (1) DPRs toxicity, in which upstream ATG-driven translation may generate poly-PG DPR as the most toxic species compared to other DPRs, and (2) RNA toxicity mediated by expanded (G3C2T)n repeat-containing transcripts. The patient-derived cells and transgenic *Drosophila* models consistently recapitulate these pathological features, underscoring their utility for investigating disease mechanisms and serving as platforms for the development and testing of potential therapeutic candidates.

As clearly delineated at Fig 10 as a working model of this study, we have characterized the effects of a series of modified (G3C2T)n repeat expansions in *Drosophila* to elucidate toxic-gain-of function mechanism underlying SCA36 pathogenesis. Our data demonstrated that both HRR and DPR contribute to neurotoxicity and serve as critical pathogenic factors in SCA36. The production and effects of DPR were verified in the SCA36 cells and *Drosophila* models (Figs 1–3). Among the different DPR products, PG-DPR exerted the most potent toxic effects (Figs 4–6). Additionally, our experiments demonstrated a positive correlation between the repeat length and severity of neurodegeneration (Fig 7), which may resemble the clinical observations that individuals with shorter pathogenic expansions tend to present with milder disease phenotypes [4]. Utilizing the tandem-STOP and codon-modified transgenes, we further confirmed the combinatorial effects of HRR and DPR on the neurotoxicity that led to SCA36 neurodegeneration (Fig 8). Taken together, the fly model of SCA36 recapitulates several key aspects of the human disorder, including locomotion disability, sensory neuron injury, and late adulthood onset disease. Our study provides a valuable tool for understanding the molecular mechanisms and a drug screening platform for potential upcoming therapies.

Unraveling the disease-underlying mechanisms of non-coding repeat expansions remains a significant challenge. The HRR-associated toxicity is considered as a crucial factor for repeat expansions disorders, since previous research identified the neurotoxic RNA foci in models of C9-ALS-(G4C2)n and SCA36-(G3C2T)n. Interestingly, while large RNA aggregation foci were observed in C9-ALS, SCA36 is characterized by smaller RNA aggregates, suggesting potential differences in aggregation dynamics and cellular impact [16,17]. Utilizing the RNA binding protein FMRP as a marker for stress granule, we observed an increased accumulation of stress granule signals both in *Drosophila* expressing DPRs (Fig 5A and 5B) and in fibroblasts derived from SCA36 patients (Fig 9D). Notably, a previous report indicated the absence of ataxin-2-labeled stress granules and TDP-43 pathology in SCA36 [17]. This discrepancy between FMRP- and ataxin-2-associated stress granules may reflect distinct stress response pathways involved in SCA36, further underscoring the complexity of RNA toxicity in the SCA36 pathogenesis. These observations also highlight a divergence in stress granule composition and downstream RNA metabolic effects between SCA36 and C9-ALS. Moreover, our series of transgenic flies carrying different repeat lengths demonstrated a positive correlation between the length of (G3C2T)n HRR and severity of neurotoxicity (Fig 7). Upon introducing these various repeat lengths in the motor neuron, significant neurodegeneration was evident (Fig 7B and 7C). This observation validated the HRR toxicity in motor neuron pathology of SCA36.

In terms of DPR toxicity, the (G3C2T)n repeat within the sense reading frame of *NOP56* potentially could generate three species of DPR: PG, AW, and GL, raising the questions regarding the individual contributions of each DPR to the disease pathogenesis. However, studies in C9-ALS demonstrated that selective DPR reduction did not yield clinical benefit [33], highlighting the disease complexity and supporting the necessity of examining the pathogenic characteristics of each DPR. The sole presence of PG-DPR in SCA36 was attributed to the uORF-mediated translation [16,26]. Furthermore, the PG-DPR appeared to be diffusely distributed in the cytoplasm of our SCA36 fibroblast (Fig 2). Similarly, a previous study had also suggested GP-DPR does not aggregate in SCA36 patient tissue. In contrast to DPR in C9-ALS, which require combination of specific DPRs for aggregation [16]. Elucidating the toxic mechanisms from individual and combined DPRs awaits further studies for SCA36.

To simultaneously evaluate the relative toxicity raised from individual DPR, we compared the effects of specific DPR in flies. The flies expressing the GL-, AW-, PG-DRPs displayed obvious neurodegenerative phenotypes in the brain, motor neuron, and sensory neurons (Figs 5 and 6). This age-associated neurodegeneration is most prominent when expressing the PG-DPR. These DPRs of 33 repeats were efficient to induce neurotoxic effects, and appeared to contribute to stress-response apoptotic activation and stress granule formation (Figs 4 and 5). It remains an open question whether these effects would be similarly or prominently observed in the patient samples and in the animals expressing the longer repeats of DPRs, or if the presence of additional contributing factors is required. Nonetheless, the partial neurotoxicity of tandem-STOP and codon-modified transgenes suggests the combinatorial effect of HRR and DPR exerts the most significant impact on the pathophysiological changes associated with SCA36 (Fig 8). These findings provide valuable insights into the complexity of the pathogenesis of SCA36, demonstrating that our multimodal approach could be utilized to investigate the therapeutic effects of potential drug targets.

Spt4 and Spt5 form a complex that facilitates RNA polymerase II in transcription elongation, thus regulates HRR transcription and DPR translation [34]. Given that Spt4/5 complex may stabilize the transcription of extended tandem repeats, Spt4 inhibition has been postulated as a potential therapeutic target for repeat expansion disorders. In fact, inhibiting Spt4 has been shown to reduce poly-GP HRR and DPR expression in animal models and iPSC-derived cortical neurons of C9-ALS-(G4C2)n [14]. Similarly, in models of Huntington’s disease, Spt4 depletion selectively reduced trinucleotide repeats mRNA and polyQ mutant huntingtin protein levels [13]. While these studies indicate selective activity at repeat expansions, other findings suggest a broader impact on global transcription [35]. Unlike in yeast, where Spt4 is non-essential, depletion of *SUPT4H1* in human cell lines has been associated with widespread transcriptional downregulation. The full extent of the global consequences resulting from downregulation of this complex remains to be determined. To assess whether disrupting Spt4 could be a viable therapeutic approach, we examined the effects of *Spt4* knockdown on neuronal function in the fly SCA36 model. Surprisingly, Spt4 inhibition not only failed to restore the locomotor deficits but exacerbated the neurotoxic effects (Fig 9A and 9B). Instead of rescuing locomotor function, administration of 6-AZA, an inhibitor of the Spt4/5 complex, worsened the phenotype upon aging (Fig 9C). Moreover, treatment with 6-AZA in patient-derived fibroblasts resulted in marked accumulation of stress granules (Fig 9D). These findings suggest that HRR and DPR toxicity may involve mechanisms beyond the Spt4-dependent pathway, leading to unanticipated toxicity. Our results emphasize the need for further research to clarify these mechanisms and guide the development of targeted therapeutic strategies.

The pathogenic role of *NOP56* loss-of-function in SCA36 remains incompletely understood. Initial studies in patient-derived lymphoblastoid cell lines reported no significant changes in *NOP56* mRNA expression [1], whereas more recent analyses of cerebellar cortex tissue revealed *NOP56* upregulation in affected individuals [16]. In contrast, other investigations using peripheral blood, iPSCs, and iPSC-derived neurons from SCA36 patients have consistently shown a marked reduction in *NOP56* transcript levels [11,36]. This inconsistency complicates efforts to definitively conclude whether *NOP56* expression is globally dysregulated in SCA36, and raises the possibility that *NOP56* loss-of-function may also contribute to disease pathogenesis. Emerging evidence suggests that *NOP56* downregulation may result from aberrant splicing and simultaneously gives rise to abnormal RNA species translating into toxic DPRs [16,37]. This may imply a dual loss-of-function and gain-of-toxicities pathomechanism in SCA36. Supporting the potential importance of *NOP56* function in the nervous system, zebrafish models have demonstrated that *NOP56* depletion impairs ribosomal RNA processing, disrupts neurodevelopment, increases motor neuron vulnerability, and dysregulates the expression of ALS-associated genes [38]. Given limitations of our current model, the relative contribution of *NOP56* loss-of-function remains to be clarified and future studies are warranted to dissect this aspect of SCA36 pathogenesis.

In conclusion, we have developed a *Drosophila* SCA36 model displaying cellular and phenotypical deficits resembling the observations in patients. Our experiments have demonstrated the synergistic effects of HRR and DPR to the complexity of SCA36 pathogenesis. Furthermore, the SCA36 flies and the patient-derived fibroblasts can be utilized to validate the potential therapeutic strategies for SCA36.

## Supporting information

S1 Fig Schematic overview of the SCA36 repeat cloning strategy. **(A)** BssHII-XhoI-digested fragment from pUAST-attB was subcloned into the pEGFP-N1 backbone to generate the intermediate construct pEGFP-N1-pUASTattB(BX) (1). Annealed oligonucleotides (#1 and #2) were inserted via NheI-XhoI, introducing five G3C2Trepeat units along with engineered restriction sites for subsequent repeat extension (2). To assess dipeptide repeat toxicity, additional ATG trinucleotides were incorporated into the oligonucleotides. Repeat length was extended by subcloning a BsaI-SacI-digested insert into a BbsI-SacI-digested vector. In the illustrated example, the repeat number was increased from 5 to 9 copies (3). This iterative cloning approach was used to generate all G3C2T repeat constructs described in this study. Final constructs were subcloned back into the pUAST-attB vector via BssHII-XhoI for subsequent Drosophila embryo injection (4). Check Materials and Methods sections for more details.
(TIFF)

S2 Fig Agarose gel analysis of restriction enzyme-digested pUAST-attB-(G3C2T)n plasmids. pUAST-attB plasmids containing the indicated number of G3C2T repeats were digested with BglII and XhoI, followed by separation on a 1.5% agarose gel. M indicates the 100 bp DNA ladder. The expected fragment sizes for constructs containing 20, 33, and 90 repeats are approximately 157 bp, 235 bp, and 577 bp, respectively.
(TIFF)

S3 Fig Validation of the *SUPT4H1* expression levels of in fibroblasts from SCA36 patients and *Spt4* knockdown efficiency of in the *Drosophila* model. Quantitative PCR (qPCR) experiments were carried out to assess *SUPT4H1* expression in human fibroblast cell lines and *Spt4* knockdown efficiency in *Drosophila*. **(A)** Fibroblast derived from two healthy controls and three independent SCA36 patients were evaluated for the expression of human *Spt4* ortholog *SUPT4H1*. The *SUPT4H1* expression levels were comparable in the SCA36 and control groups. **(B)** *Spt4* knockdown efficiency of the *Spt4* RNAi line was estimated at approximately 34% by qPCR.
(TIFF)
