# Supplementary figures and images for: A fly model of SCA36 reveals combinatorial neurotoxicity of hexanucleotide and dipeptide repeats
Source: PLoS Genet. 2025 Dec 3;21(12):e1011954. doi: 10.1371/journal.pgen.1011954 (PMC12674567; doi:10.1371/journal.pgen.1011954)

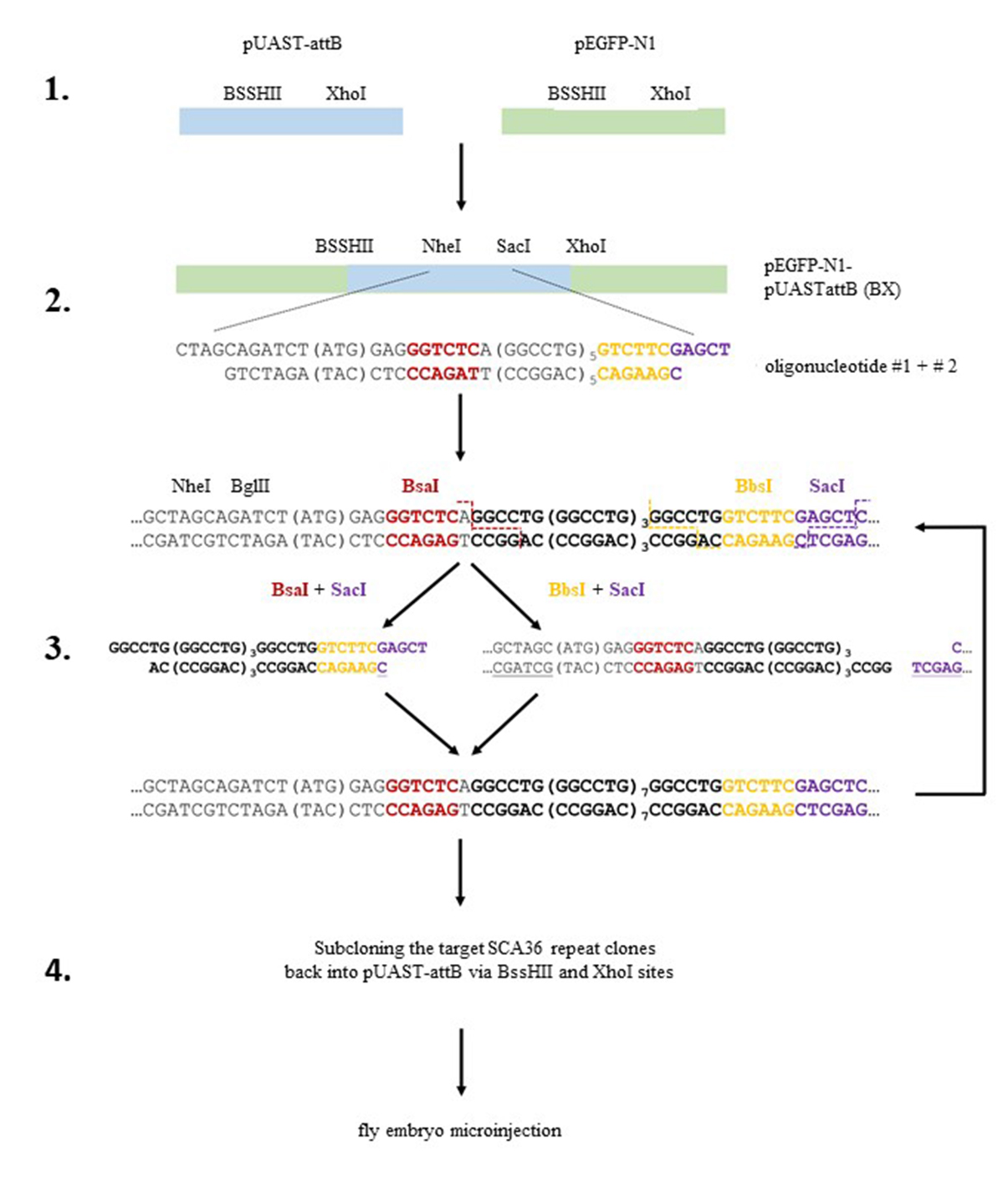

Supplement: S1 Fig — (A) BssHII-XhoI-digested fragment from pUAST-attB was subcloned into the pEGFP-N1 backbone to generate the intermediate construct pEGFP-N1-pUASTattB(BX) (1). Annealed oligonucleotides (#1 and #2) were inserted via NheI-XhoI, introducing five G3C2Trepeat units along with engineered restriction sites for subsequent repeat extension (2). To assess dipeptide repeat toxicity, additional ATG trinucleotides were incorporated into the oligonucleotides. Repeat length was extended by subcloning a BsaI-SacI-digested insert into a BbsI-SacI-digested vector. In the illustrated example, the repeat number was increased from 5 to 9 copies (3). This iterative cloning approach was used to generate all G3C2T repeat constructs described in this study. Final constructs were subcloned back into the pUAST-attB vector via BssHII-XhoI for subsequent Drosophila embryo injection (4). Check Materials and Methods sections for more details. (TIFF) [file pgen.1011954.s001.tiff]

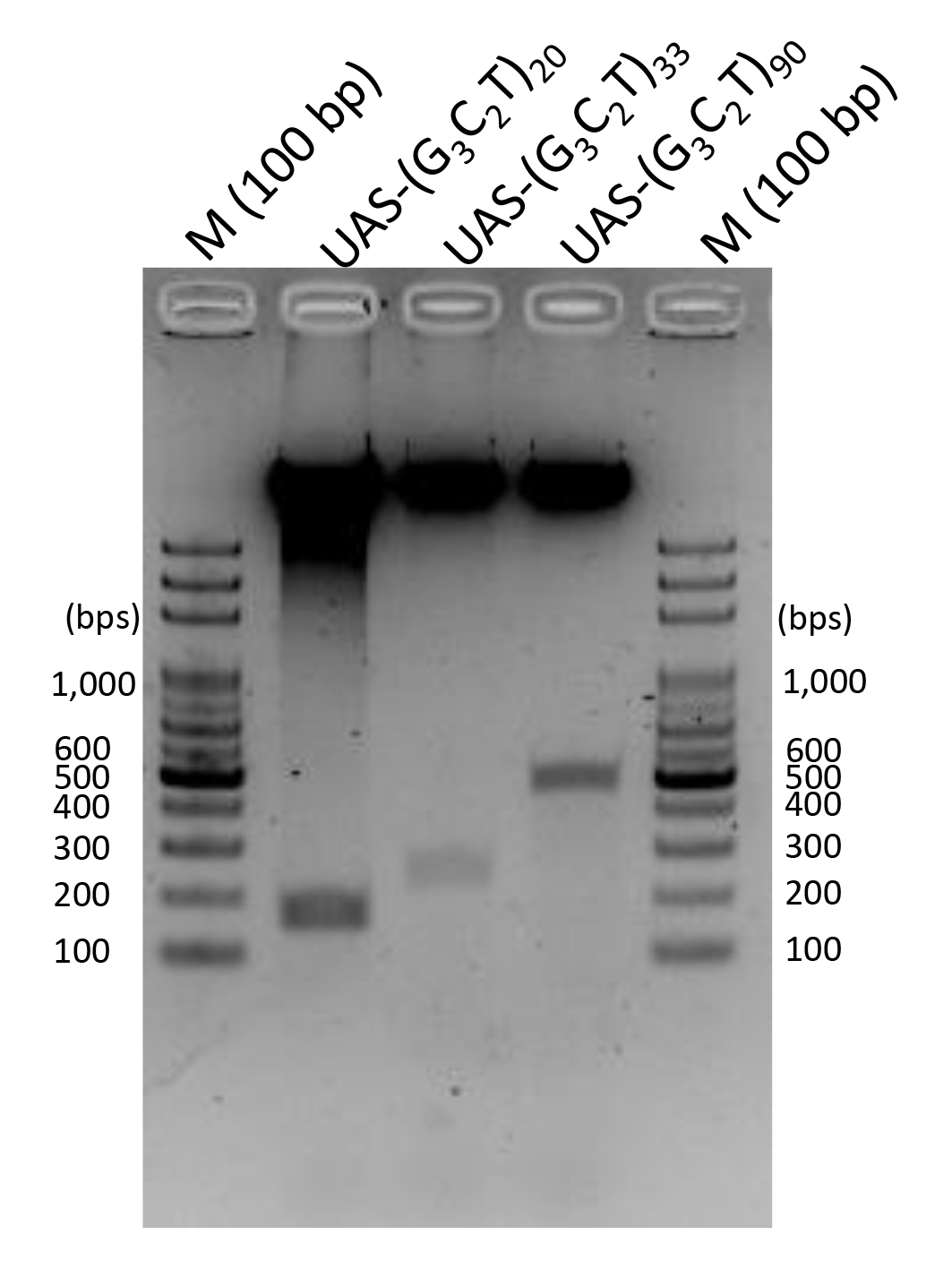

Supplement: S2 Fig — pUAST-attB plasmids containing the indicated number of G3C2T repeats were digested with BglII and XhoI, followed by separation on a 1.5% agarose gel. M indicates the 100 bp DNA ladder. The expected fragment sizes for constructs containing 20, 33, and 90 repeats are approximately 157 bp, 235 bp, and 577 bp, respectively. (TIFF) [file pgen.1011954.s002.tiff]

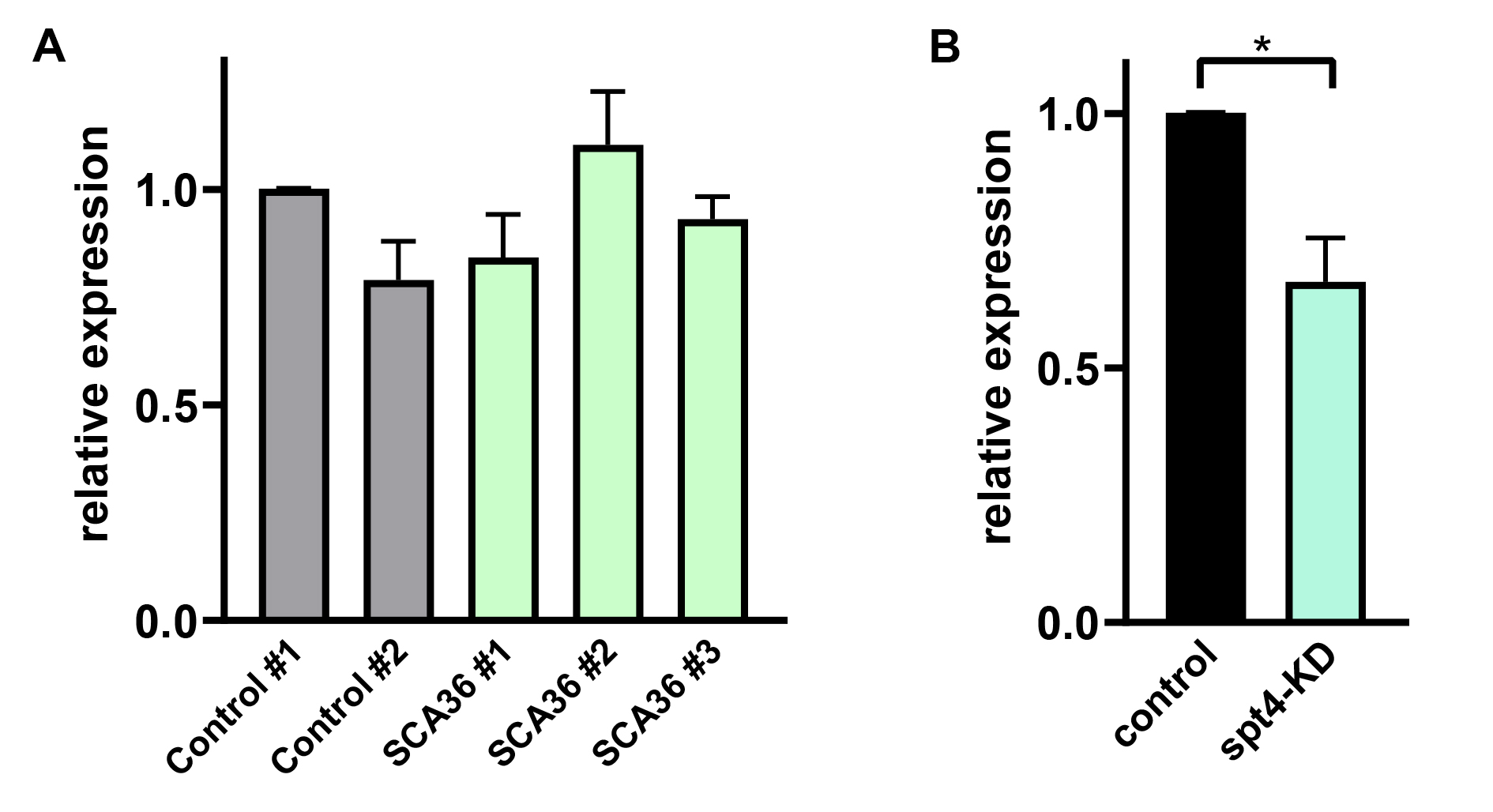

Supplement: S3 Fig — Quantitative PCR (qPCR) experiments were carried out to assess SUPT4H1 expression in human fibroblast cell lines and Spt4 knockdown efficiency in Drosophila. (A) Fibroblast derived from two healthy controls and three independent SCA36 patients were evaluated for the expression of human Spt4 ortholog SUPT4H1. The SUPT4H1 expression levels were comparable in the SCA36 and control groups. (B) Spt4 knockdown efficiency of the Spt4 RNAi line was estimated at approximately 34% by qPCR. (TIFF) [file pgen.1011954.s003.tiff]
